# Trusting and staying with AI doctors: cognitive and emotional pathways driving continuance intention toward AI healthcare chatbots

**DOI:** 10.3389/fpubh.2026.1956063

**Published:** 2026-09-09

**Authors:** Zhanyou Wang, Haoran Han, Dongmei Han, Liang Ma, Feifei Hao

**Affiliations:** 1 School of Business Administration, Shandong Management University, Jinan, China; 2 School of Labor Relations, Shandong Management University, Jinan, China; 3 School of Information Engineering, Shandong Management University, Jinan, China; 4 School of Management Science and Engineering, Shandong University of Finance and Economics, Jinan, China; 5 College of Traditional Chinese Medicine, Shandong University of Traditional Chinese Medicine, Jinan, China

**Keywords:** AI doctor use, cognitive trust, continuance intention, emotional attachment, technology anxiety

## Abstract

**Introduction:**

AI-powered medical chatbots are increasingly used in healthcare, yet the mechanisms underlying users' continued engagement remain unclear. This study examines how AI use influences continuance intention through cognitive trust and emotional attachment and whether technology anxiety differentially moderates these pathways.

**Methods:**

Data were collected from 233 users of AI medical services in China. The proposed model was tested using partial least squares structural equation modeling (PLS-SEM) and bootstrapping in SmartPLS 4.0.

**Results:**

AI use positively influences both cognitive trust and emotional attachment, which in turn enhance continuance intention. Both mechanisms significantly mediate the relationship between AI use and continuance intention, with emotional attachment showing a stronger mediating effect. Technology anxiety significantly moderates the AI use–cognitive trust relationship but not the AI use–emotional attachment relationship, revealing an asymmetric moderating pattern.

**Discussion:**

This study extends cognitive-affective processing theory by revealing dual cognitive and emotional pathways linking AI use to continuance intention. It further identifies technology anxiety as a boundary condition that selectively shapes the cognitive pathway while leaving the emotional pathway relatively unaffected. The findings offer implications for designing anxiety-sensitive AI healthcare services and differentiated user engagement strategies.

## 1 Introduction

Artificial intelligence (AI)-powered medical Chatbots, commonly referred to as AI doctors, have emerged as transformative tools in modern healthcare, providing accessible and immediate health information and diagnostic suggestions to users worldwide. Despite their growing prevalence, ensuring that users continue to engage with these AI medical services over time remains a critical challenge for healthcare providers and technology developers. Recent industry reports indicate that the global AI healthcare market is projected to reach $188 billion by 2030 (1), yet user retention rates for AI medical Chatbots remain suboptimal, with approximately 40% of users discontinuing use after initial trials. As AI technologies rapidly evolve, incorporating advanced features such as natural language processing and personalized health recommendations, understanding the psychological mechanisms that drive sustained user engagement becomes increasingly urgent. This study develops and tests a comprehensive research model examining how AI use is associated with continuance intention through the dual mediating pathways of cognitive trust and emotional attachment, while also investigating the moderating role of technology anxiety. In this study, AI use refers not merely to the frequency or duration of interacting with AI healthcare Chatbots, but to the extent to which users actively employ AI doctors for obtaining health information, supporting self-diagnosis, and assisting healthcare-related decision-making. Thus, AI use captures users' behavioral engagement with AI healthcare services in practical medical contexts, reflecting both functional utilization and interaction involvement. By doing so, this research aims to provide both theoretical advancements and practical guidance for designing more effective and user-centered AI healthcare services.

While prior research has extensively examined technology acceptance and continuance using cognitive-based models such as the Technology Acceptance Model (TAM) and Expectation Confirmation Theory (ECT), these frameworks largely emphasize rational evaluations of usefulness and satisfaction, often overlooking the emotional dimension of human-technology interaction. Unlike traditional healthcare technologies that primarily function as information-delivery systems, AI healthcare Chatbots possess both informational and relational characteristics. On the one hand, AI Chatbots provide users with diagnostic suggestions, medical explanations, and personalized recommendations, requiring users to cognitively evaluate system competence, accuracy, and reliability. On the other hand, conversational interfaces, immediate feedback, and personalized interactions create social presence and emotional experiences, encouraging users to form affective connections with AI systems. Therefore, AI use simultaneously activates cognitive and affective processing because AI healthcare Chatbots serve not only as decision-support tools but also as interactive social actors. Drawing upon cognitive-affective processing theory, which posits that both cognitive and affective mechanisms jointly shape human responses, this study argues that understanding user continuance requires simultaneous consideration of both rational trust and emotional bonds. In practice, users of AI medical services frequently report both cognitive assessments of system reliability and emotional experiences of closeness or comfort during interactions, suggesting that both pathways are relevant in healthcare contexts. However, existing literature has largely treated trust as a unidimensional construct (2) and has not systematically distinguished between cognitive trust (competence-based rational evaluation) and emotional attachment (affective, relationship-based bond) as distinct mediating mechanisms. Although cognitive trust and emotional attachment are both related to users' positive perceptions of AI healthcare Chatbots, they originate from fundamentally different psychological processes. Cognitive trust reflects a competence-based judgment process in which users evaluate whether an AI system possesses sufficient capability, reliability, and expertise to fulfill healthcare-related tasks. In contrast, emotional attachment represents a relational bonding process through which users develop feelings of closeness, familiarity, and psychological connection based on repeated interactions. Therefore, cognitive trust answers the question of “Can this AI doctor provide reliable healthcare services?”, whereas emotional attachment addresses “Do I feel connected and comfortable interacting with this AI doctor?” (3). This theoretical gap is particularly significant because cognitive and affective pathways may operate differently and respond to contextual factors in unique ways, yet prior studies have rarely examined how these two pathways function simultaneously and comparatively within the same model. Understanding these distinct mechanisms is crucial for developing targeted interventions that can effectively promote sustained user engagement with AI healthcare services (4). Furthermore, while technology anxiety has been recognized as a barrier to initial technology adoption (5), limited attention has been paid to how it differentially shapes the ongoing psychological processes that sustain use, particularly whether it affects cognitive and emotional pathways differently. In practice, many potential users of AI medical services report feelings of unease or apprehension about using AI for health-related decisions (6), yet some still develop emotional connections with these systems over time, suggesting that anxiety may not uniformly hinder all psychological pathways. Building on cognitive-affective processing theory (7), which suggests that affective responses can operate independently of cognitive appraisals under conditions of uncertainty or stress, this study proposes that technology anxiety may selectively disrupt cognitive evaluations while leaving emotional bonding relatively intact. Specifically, no prior research has empirically examined whether technology anxiety differentially moderates the cognitive vs. emotional pathways in the context of AI healthcare services (8), leaving a significant gap in understanding how user apprehensions shape distinct psychological mechanisms. Addressing this gap is critically important because it would enable healthcare providers and system designers to develop targeted, differentiated intervention strategies that address the unique vulnerabilities of each psychological pathway, rather than applying one-size-fits-all approaches to user engagement. This study addresses these research gaps by investigating:

RQ1: How AI use is associated with continuance intention through cognitive trust and emotional attachment as dual mediators?

RQ2: Whether technology anxiety differentially moderates the relationships between AI use and these two mediating pathways?

To achieve this, we conducted a survey-based quantitative study involving 233 users of AI medical services in China. Data were collected through a structured questionnaire and analyzed using structural equation modeling (SEM) with SmartPLS 4.0 software to empirically test the proposed research model and hypotheses. Our findings reveal that both cognitive trust and emotional attachment significantly mediate the relationship between AI use and continuance intention, with emotional attachment exerting a stronger total effect, and that technology anxiety significantly moderates the cognitive pathway but not the emotional pathway, revealing an asymmetric moderating pattern. This study makes several important theoretical contributions to the literature on AI adoption, human-computer interaction, and technology continuance in healthcare contexts. First, while previous research has predominantly relied on cognitive-based models, this study extends cognitive-affective processing theory by integrating both cognitive and emotional pathways into a unified framework, revealing that emotional attachment exerts a stronger influence on continuance intention than cognitive trust. Second, whereas prior research has largely treated trust as a unidimensional construct, this study distinguishes between cognitive trust and emotional attachment as distinct mediating mechanisms, uncovering their complementary yet differentiated roles and demonstrating that the emotional pathway exhibits greater resilience to technology anxiety. Third, while existing literature has examined technology anxiety as a barrier to initial adoption, this study identifies it as a critical boundary condition that selectively disrupts cognitive pathways while leaving emotional pathways relatively intact, revealing a more nuanced pattern of how user apprehensions shape ongoing engagement with AI healthcare services.

## 2 Theoretical background

### 2.1 Continuance intention and AI doctor use

The concept of continuance intention originates from expectation-confirmation theory, which refers to the extent to which users form repeat usage intentions based on their satisfaction with prior experiences (9). Early research primarily examined this concept in the context of general information systems, confirming that technology characteristics, user characteristics, service quality, and trust significantly influence continuance intention, framing the process as a result of rational cognitive evaluation. Over the past decade, the rapid development of artificial intelligence technologies, particularly conversational AI and AI-powered Chatbots, has fundamentally transformed healthcare service delivery (10), creating new paradigms for human-technology interaction (11). In this emerging context, continuance intention is no longer determined solely by rational technology evaluation but is also shaped by interpersonal-like dynamics. Meta-analytic evidence reveals that in text-only interactions, AI Chatbots are perceived as more empathetic than human healthcare professionals (12).

Existing literature has identified various antecedents of continuance intention in AI contexts, confirming that trust perception is positively associated with technology adoption, and that both cognitive empowerment and emotional empowerment significantly affect users‘ usage intentions (13). However, a critical gap remains: prior studies have largely treated trust as a unidimensional construct, failing to distinguish between its cognitive and affective dimensions. Although information systems scholars have extensively explored the multidimensional nature of trust, most empirical studies still operationalize trust as a single aggregated construct, obscuring the distinct mechanisms through which different trust dimensions operate (14). This limitation is particularly salient in healthcare contexts. Research demonstrates that cognitive trust and affective trust play fundamentally different roles in shaping users' acceptance of AI-based interventions. For instance, studies on trust repair in AI doctors reveal that emotional support is associated with trust restoration following service failures, suggesting that affective mechanisms can operate independently of cognitive appraisals (15). Meanwhile, technology anxiety negatively associated with attitudes toward use and usage intentions, while moderating the impact of perceived credibility on perceived usefulness (12). Patient perspectives further emphasize this point, showing that trust in healthcare systems and providers is positively associated with expectations of AI, highlighting the importance of relational factors (16). Addressing these theoretical gaps, this study proposes and tests a dual-pathway model: AI use is associated with continuance intention through two mediating mechanisms—cognitive trust and emotional attachment—while examining whether technology anxiety differentially moderates these two psychological pathways. This theoretical nuance addresses critical gaps in understanding the complex psychological processes underlying sustained engagement with AI healthcare services (Table 1).

**Table 1:** Summary of empirical studies on the relationship between AI use and continuance intention.

Study	Use dimension	Mediator variable	Moderator variable	Dependent variable	Conclusion
Kyung et al. (3)	AI-based preventive health interventions	Cognitive trust, Affective trust	Human expert opinion, Algorithmic transparency	Acceptance of AI interventions	The effectiveness of AI in preventive health interventions can be improved, but user acceptance of AI is lower than that of human experts. A lack of affective trust (rather than cognitive trust) is the decisive factor; combining human expert opinions, increasing algorithmic transparency, or emphasizing AI's warmth and care can improve acceptance.
Wu et al. (6)	Al Technology Introduction	AI creates new interaction paradigms, requiring a reconceptualization of Continuance	N/A	Failure to distinguish cognitive and affective pathways	Although AI has established new interaction paradigms that demand a renewed understanding of continuance usage, existing studies inadequately differentiate between cognitive and affective mechanisms underlying users' continued AI adoption.
Chen et al. (19)	Generative AI post-adoption use	Trust (positive), Fear (negative)	Transparency	Extended use, Avoidance	GAI doctors with internal attribution, emotional support, or anthropomorphism produce higher levels of trust repair. The effect of social support on behavioral intention is fully mediated by trust repair.
Gupta and Banerjee (68)	AI integration at work	Technostress	Tech-savviness	Quiet quitting intention	AI anxiety influences employees' quiet quitting intention through technostress. Tech-savviness moderates the relationship between AI anxiety and technostress.
Nan et al. (69)	Generative AI (ChatGPT) use	Information quality; System quality; Privacy concerns; Perceived innovativeness	N/A	Continuance intention to use and recommend	Information quality, system quality, privacy concerns, and perceived innovativeness affect users' continuance intention and recommendation intention toward generative AI (ChatGPT).
Lee et al. (70)	AI-powered Chatbot use (performance-approach vs. performance-avoidance goal orientation)	Novelty value perception; Hedonic attitude; Utilitarian cognitive attitudes; Performance expectancy	N/A	Continuance intention	Performance-approach and performance-avoidance goal orientations influence continuance intention toward AI Chatbots through novelty value perception, hedonic attitude, utilitarian cognitive attitude, and performance expectancy.
Kai et al. (30)	AI anxiety dimensions (learning, sociotechnical blindness, job displacement)	Performance expectancy; Effort expectancy; Social influence	Disciplinary background; AI self-efficacy	GAI adoption intention	AI anxiety exerts a significant “double-edged sword” effect on the adoption intention of generative AI: job replacement anxiety comprehensively inhibits adoption; socio-technical blindness anxiety positively promotes effort expectancy and adoption intention while negatively affecting performance expectancy.
Arora et al. (7)	AI and robotics adoption in healthcare	Utilitarian value; Control beliefs; Anthropomorphism; Perceived risks; Ethical dilemmas	Interaction comfort	Adoption intention	Practical value, control belief, and anthropomorphism positively influence repeated adoption intention, whereas perceived risk and ethical dilemmas exert negative impacts. Interaction comfort moderates the relationship between adoption intention and work-life quality.
Zhang et al. (71)	Perceived interactivity of AI conversational agents	Trust; Social presence	N/A	Continuance usage intention	Technical dimensions such as controllability and responsiveness influence continuous use through trust; affective interaction dimensions including communication, personalization, and entertainment influence continuous use through social presence.
Wu et al. (6)	Conversational AI use in digital counseling	Dispositional trust; Anxious attachment style	N/A	Adoption intention	Trait trust and attachment anxiety positively predict the adoption intention of AI psychological counseling; anxious attachment style indirectly affects adoption intention by weakening trust.

### 2.2 Cognitive-affective processing theory

Cognitive-affective processing theory originates from the integration of cognitive and affective psychology, positing that human judgment and behavior emerge from the interplay between two distinct yet interacting systems: a cognitive system responsible for deliberate, analytical information processing, and an affective system that operates through automatic, experiential mechanisms (17). Importantly, cognitive trust and emotional attachment should not be viewed as two overlapping dimensions of trust or relationship quality. Cognitive trust is rooted in epistemic evaluation, where users seek confidence regarding whether AI doctors are competent and reliable. Emotional attachment, however, originates from relational experience, where users seek psychological comfort and interpersonal-like connection. Although both mechanisms may contribute to continuance intention, they satisfy different psychological needs: cognitive trust reduces uncertainty, whereas emotional attachment fulfills users' needs for social connection and emotional reassurance.

This theoretical lens is particularly well-suited for examining continuance intention in AI healthcare services for three reasons: First, AI medical interactions inherently involve both rational evaluation (e.g., assessing diagnostic accuracy) and emotional experience (e.g., feeling understood or cared for), mirroring the dual-system structure of the theory. This aligns with findings that both cognitive empowerment and emotional empowerment significantly predict users' intention to use AI-based medical consultations (18). Second, the theory explicitly acknowledges that affective processes can operate independently of cognitive appraisals, which is crucial for understanding why emotional attachment may persist even when cognitive trust is undermined—a pattern observed in trust repair studies where emotional support restores trust after service failures (19). Third, the theory provides a framework for explaining differential susceptibility to contextual factors. This is particularly relevant for examining how technology anxiety may selectively disrupt the cognitive pathway while leaving the affective pathway relatively intact—a theoretical nuance that prior research, which has largely treated trust as unidimensional, has failed to capture (20). Critically, the cognitive system is resource-dependent and vulnerable to affective disruptions, whereas the affective system operates automatically and is more resilient to cognitive interference.

Extending cognitive-affective processing theory to AI healthcare contexts, we argue that AI use represents a unique technological experience that simultaneously provides cognitive resources and affective experiences. Consistent with recent developments in information systems research, digital technologies create value not only through functional capabilities but also through interaction quality, user experience, and relational engagement. The updated IS success perspective emphasizes that system quality and interaction quality jointly shape users' perceptions and behavioral responses, suggesting that technology adoption involves both instrumental evaluation and experiential processing (21–23). Accordingly, AI use activates cognitive processing by enabling users to accumulate evidence about AI competence, reliability, and usefulness. Meanwhile, repeated conversational interactions activate affective processing by generating feelings of familiarity, support, and psychological connection. These two processes are complementary but not interchangeable: cognitive processing answers whether AI can provide reliable healthcare services, whereas affective processing concerns whether users feel comfortable and connected during interactions. From the cognitive-affective perspective, technology anxiety represents a negative emotional state that consumes cognitive resources and disrupts deliberate evaluation processes (24). Therefore, users experiencing higher anxiety may find it more difficult to systematically assess AI competence, weakening the formation of cognitive trust. However, emotional attachment is developed through accumulated interaction experiences and social presence, which are relatively automatic and less dependent on conscious evaluation. Thus, technology anxiety may have asymmetric effects on cognitive and affective pathways.

### 2.3 The unique role of emotional attachment in AI healthcare contexts

Unlike general-purpose AI applications (e.g., entertainment or productivity tools), AI-powered medical Chatbots operate in a context characterized by emotional vulnerability, uncertainty about health outcomes, and a strong need for reassurance. In such settings, users may develop emotional attachment not to a brand or a physical product, but to the interaction experience and the perceived social presence of the AI entity. Four interrelated theoretical streams help explain this phenomenon.

First, the Computers Are Social Actors (CASA) paradigm posits that humans automatically apply social rules and expectations to computers, especially when the technology exhibits human-like characteristics (25). This effect is amplified in healthcare because patients' desire for empathy and social support heightens their sensitivity to social cues. Second, social presence—the sense of being with another sentient being—can be evoked by conversational agents through empathetic language, personalized memory, and responsive feedback. Higher social presence leads to stronger emotional bonds, as users perceive the AI as a companion rather than a mere tool. Third, anthropomorphism, the attribution of human traits to non-human entities, further facilitates emotional attachment. When an AI doctor uses a warm tone, remembers past interactions, or expresses concern, users instinctively treat it as a social partner (26). Fourth, these factors together foster parasocial interaction—a one-sided psychological relationship where users feel closeness and trust toward a media persona or an AI (27). In the context of AI doctors, parasocial interaction transforms a purely functional service into an affectively charged experience.

Recent meta-analytic evidence reinforces this view: AI Chatbots are perceived as more empathic than human healthcare professionals in text-only interactions (12). This empathy perception directly fuels parasocial bonding and emotional attachment. Consequently, in AI healthcare services, emotional attachment is grounded in the user' s accumulated positive interactions with the AI, the sense of being understood, and the feeling of a shared relationship—not in brand loyalty or product evaluation. This contrasts sharply with cognitive trust, which relies on rational assessment of competence and reliability. Thus, the two constructs are theoretically and empirically distinct, and emotional attachment is expected to exert a stronger influence on continuance intention in the affect-laden healthcare context. This distinctive property—that emotional attachment is less susceptible to disruption by negative affective states such as technology anxiety—is what we term affective resilience. It forms the theoretical foundation for understanding why emotional bonds with AI doctors can persist even when cognitive trust is undermined.

## 3 Hypothesis development

### 3.1 AI use, cognitive trust, and emotional attachment

Cognitive trust refers to users' competence-based rational evaluation that an AI doctor possesses the necessary reliability, diagnostic capability, and professional knowledge to provide accurate and effective healthcare services. It reflects a rational judgment process in which users assess whether the AI system is capable of performing expected medical functions, such as interpreting health information, generating appropriate recommendations, and providing reliable responses. In contrast, emotional attachment represents an affective bond developed through repeated interactions, characterized by feelings of familiarity, psychological closeness, comfort, and habitual reliance on the AI healthcare service. Unlike cognitive trust, emotional attachment does not require users to continuously verify AI's technical competence. Instead, it develops through accumulated interaction experiences, perceived social presence, and emotional reassurance. Therefore, emotional attachment cannot be reduced to affective trust because it reflects a relationship-maintenance mechanism rather than merely a positive evaluation of AI's characteristics. Importantly, emotional attachment does not imply that users perceive AI as a human substitute; rather, it reflects users' emotional connection with the interaction experience, including feeling understood, supported, and accompanied during health-related decision-making processes (28). The distinction between cognitive trust and emotional attachment is particularly important in AI healthcare contexts. Unlike conventional digital services, healthcare decisions often involve uncertainty, vulnerability, and personal concerns, making users' continued engagement dependent not only on whether an AI system is perceived as competent but also on whether the interaction experience satisfies users' emotional needs. Cognitive trust provides the rational foundation for users to accept AI-generated medical information, whereas emotional attachment represents the affective pathway through which users develop psychological dependence and willingness to maintain long-term interactions. Therefore, AI healthcare continuance is unlikely to be explained by a purely cognitive evaluation process; instead, it emerges from the integration of rational confidence and emotional connection. AI use serves as an important antecedent of both cognitive trust and emotional attachment. First, repeated interaction with AI doctors enables users to accumulate direct experience regarding system performance, response quality, and medical information accuracy. Importantly, AI use in this study reflects meaningful interaction rather than mere exposure frequency. Frequent and purposeful use allows users to observe AI doctors' response quality, reliability, and usefulness, thereby facilitating cognitive trust formation. Meanwhile, continuous engagement with conversational AI creates accumulated relational experiences, including familiarity, comfort, and perceived support, which foster emotional attachment. According to the familiarity-based trust perspective, continuous exposure and successful interactions reduce uncertainty and allow users to form more stable competence-based evaluations of the technology (19). As users repeatedly observe the AI doctor's ability to provide timely and useful health-related responses, they are more likely to perceive the system as reliable and trustworthy, thereby strengthening cognitive trust.

Second, frequent AI doctor use may gradually facilitate emotional attachment through repeated and personalized interactions. Unlike traditional healthcare technologies that mainly provide functional support, AI Chatbots can engage users through conversational interfaces, immediate responses, and personalized communication. These interaction characteristics may create a sense of responsiveness and social presence, allowing users to experience psychological comfort and relational connection. Particularly in healthcare scenarios, where individuals may experience uncertainty, anxiety, or concerns about their health conditions, AI-based interactions can provide continuous accessibility and emotional reassurance, thereby fostering emotional attachment. Both cognitive trust and emotional attachment are expected to promote continuance intention, but through different psychological mechanisms. Cognitive trust encourages continued use because users believe that AI doctors are capable of delivering valuable and reliable healthcare services. When users perceive AI systems as accurate and competent, they are more willing to rely on AI-generated recommendations and continue interacting with the service. Emotional attachment, however, promotes continuance intention through an affective mechanism. Users who develop psychological closeness and habitual reliance on AI healthcare services are more likely to maintain interaction because the technology becomes integrated into their health management routines and emotional experiences (26, 29). Furthermore, emotional attachment may play an especially important role in AI healthcare contexts. Health-related decisions often involve emotional vulnerability, uncertainty, and the need for reassurance. Compared with purely functional evaluations, emotional attachment may provide users with a sense of security and continuity during healthcare interactions. Therefore, although technology anxiety may weaken users' confidence in AI capabilities, emotionally attached users may remain motivated to continue using AI healthcare services because their reliance is rooted not only in perceived competence but also in accumulated interaction experiences and affective connections (12, 26). Accordingly, this study proposes the following hypotheses:

H1: AI doctor use has a positive effect on cognitive trust.

H2: AI doctor use has a positive effect on emotional attachment.

H3: Cognitive trust has a positive effect on continuance intention.

H4: Emotional attachment has a positive effect on continuance intention.

### 3.2 The moderating role of technology anxiety

Technology anxiety captures the apprehension and discomfort users experience when confronted with new technologies (30, 31). According to cognitive-affective processing theory, human decision-making involves two distinct processing modes: a cognitive system for deliberate, analytical evaluation, and an affective system for automatic, habitual, and experiential processing (32). These two systems are differentially affected by negative affective states such as anxiety. Cognitive trust—a competence-based rational evaluation—relies heavily on analytical processing. It requires users to consciously assess the AI doctor's reliability, accuracy, and professional knowledge. This effortful, resource-intensive processing is highly vulnerable to disruption by technology anxiety, which consumes attentional capacity and induces systematic biases. In contrast, emotional attachment develops through habitual and experiential processing. It emerges from repeated positive interactions, feelings of being understood, and automatic affective responses—none of which require deliberate cognitive effort. Therefore, emotional attachment is less susceptible to interference from anxiety. Based on this theoretical distinction, we hypothesize that technology anxiety will selectively weaken the cognitive pathway while leaving the affective pathway relatively intact.

For users with high technology anxiety, the tension and unease triggered during interactions with an AI doctor disrupt their capacity for rational, systematic evaluation, thereby impeding the formation of cognitive trust. Conversely, users with low anxiety can engage in smooth human-AI interaction, facilitating rational appraisal. Empirical findings confirm this asymmetric pattern: technology anxiety significantly dampens the positive effect of AI use on cognitive trust (β = −0.131, *p* < 0.05) but does not significantly moderate the relationship between AI use and emotional attachment (β = 0.023, *p* > 0.05). Furthermore, the asymmetric influence of technology anxiety can be explained by the different formation mechanisms of cognitive trust and emotional attachment. Cognitive trust represents an ongoing evaluation of AI competence and therefore remains sensitive to negative psychological states that interfere with information processing. Emotional attachment, however, reflects accumulated relational experiences and emotional connections, which may persist even when users experience anxiety toward the technology itself. Thus, we propose:

H5: Technology anxiety negatively moderates the relationship between AI use and cognitive trust, such that the positive effect of AI use on cognitive trust weakens as technology anxiety increases.

H6: Technology anxiety negatively moderates the relationship between AI use and emotional attachment, such that the positive effect of AI use on emotional attachment weakens as technology anxiety increases.

**Figure 1:**
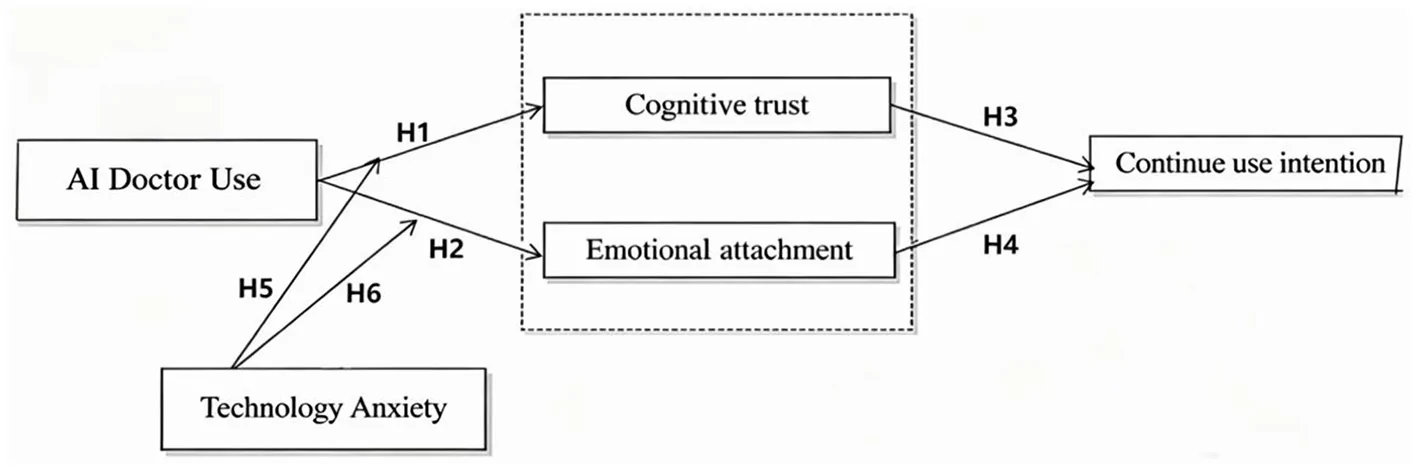
Research model.

Drawing upon cognitive-affective processing theory, we proposed our model (Figure 1).

## 4 Research methods

### 4.1 Measures

In this study, a questionnaire was used to collect data. Each measurement item was drawn from established scales in prior literature (see Table 2). Specifically, AI doctor use (AU) was measured with four items adapted from Dwivedi et al. (33) and Meuter et al. (34). Technology Anxiety (TA) was measured with four items, which were adapted from Meuter et al. (34) and Tarafdar et al. (35). Cognitive Trust (CT) was measured with four items revised from McKnight et al. (36) and Shang et al. (37). Emotional Attachment (EA) was measured with four items, which were revised from Kasturiratna and Hartanto (38) and Cheng and Yu (39). Finally, Continuance Intention (CUI) was measured with four items adapted from Bhattacherjee (9) and Venkatesh et al. (40). All of the above items were scored on a seven-point Likert scale, ranging from 1 (“strongly disagree”) to 7 (“strongly agree”). To ensure translation accuracy and contextual appropriateness, three bilingual experts in the field of information systems whose native language is Chinese were invited to revise the difficult statements in the questionnaire items. Furthermore, to ensure the reliability and validity of the items in the survey, a pilot test was conducted. The results of the pilot test showed that the measurement items were adequate for further implementation.

**Table 2:** Measurement items.

Constructs	Items measured
Medical knowledge level (72, 73)	MKL1. I possess adequate medical knowledge to understand the diagnostic advice provided by the AI doctor.
	MKL2. My medical knowledge enables me to judge whether the recommendations given by the AI doctor are reasonable.
	MKL3. I believe I have a good understanding of common diseases and treatment methods.
	MKL4. My medical knowledge makes it easier for me to interact effectively with the AI doctor.
AI doctor use (AU) (33, 34)	AU1. I frequently use AI doctor Chatbots to obtain health information.
	AU2. I use AI doctor tools in my daily life to assist with self-diagnosis.
	AU3. AI doctors are one of the important ways for me to obtain medical advice.
	AU4. I consider the advice provided by AI doctors in my health-related decisions.
Technology anxiety (TA) (34, 35)	TA1. Using AI doctors makes me feel nervous.
	TA2. I feel uneasy when encountering complex problems.
	TA3. I worry that I will not be able to use the AI doctor system correctly.
	TA4. I often feel anxious or concerned when interacting with AI doctors.
Cognitive trust (CT) (36, 37)	CT1. I believe the information provided by AI doctors is reliable.
	CT2. I think AI doctors possess professional knowledge to answer medical questions.
	CT3. I trust that AI doctors can provide reasonable health advice.
	CT4. I feel that the services provided by AI doctors are highly credible.
Emotional attachment (EA) (38, 39)	EA1. I have developed a sense of closeness to AI doctors.
	EA2. I feel that using AI doctors has become a habitual dependency.
	EA3. I develop a sense of emotional reliance when interacting with AI doctors.
	EA4. I have positive feelings toward AI doctors.
Continuance intention (CUI) (9, 40)	CUI1. I plan to continue using AI doctors as a source of health information.
	CUI2. Even if other options are available, I am still willing to use AI doctors.
	CUI3. I will continue to use AI doctors in the long term.
	CUI4. I will recommend the use of AI doctors to others.

### 4.2 Data collection

China provides a particularly appropriate context for examining users' cognitive and emotional responses toward AI healthcare Chatbots. As the world's largest healthcare market by population, China faces persistent challenges related to uneven distribution of medical resources, increasing healthcare demands, and pressure to improve service accessibility. In response, AI-enabled healthcare technologies, including medical Chatbots, intelligent consultation systems, and AI-assisted health management platforms, have been increasingly integrated into healthcare services to provide users with convenient and continuous access to medical information and preliminary health guidance. Unlike traditional digital health applications that mainly serve as information repositories, AI healthcare Chatbots in China are characterized by interactive conversational capabilities, personalized responses, and continuous user engagement, making them suitable for examining both functional evaluations and relational experiences. Moreover, the widespread adoption of mobile internet and digital healthcare platforms has created a large user base with increasing exposure to AI-mediated medical services. Through repeated interactions with AI healthcare Chatbots, users can accumulate experiences regarding system competence, reliability, and usefulness, thereby forming cognitive trust. Meanwhile, conversational interactions, personalized feedback, and perceived social presence may generate feelings of familiarity and emotional connection, facilitating emotional attachment. Therefore, the Chinese AI healthcare context provides a theoretically meaningful setting for investigating how AI use simultaneously activates cognitive and affective mechanisms underlying continuance intention.

The survey respondents were actual users of AI medical Chatbots in China. To ensure that participants had sufficient experience with AI healthcare services, respondents were required to have previously interacted with AI medical Chatbots for health-related purposes before completing the questionnaire. A screening question was first used to identify qualified respondents. Specifically, participants were asked whether they had previously used AI medical Chatbots for obtaining health information, symptom checking, or healthcare consultation. Only respondents with prior usage experience were allowed to proceed with the questionnaire. The survey respondents were actual or potential users of AI medical services, recruited from various age groups across different regions of China. Data were collected using a professional online survey platform in China, which has been widely adopted by researchers and is known for its rigorous data quality control (41–43). To improve data quality, the platform employs several mechanisms, including identity verification to prevent bot registrations, sample balancing controls, and AI-driven anti-fraud models to detect and remove low-quality participants (44). Following questionnaire design, a web-based link was distributed to a pre-screened panel of users on the platform, and respondents who completed the survey received a small monetary reward. A total of 233 valid responses were retained for subsequent analysis after removing questionnaires with missing values or uniform answer patterns. These respondents had direct interaction experience with AI healthcare Chatbots, enabling them to evaluate both the functional competence and relational experience of AI doctors, which are central to the proposed cognitive trust and emotional attachment mechanisms.

Although the sample includes a relatively high proportion of university students (67.38%), this demographic is particularly relevant for studying AI medical service continuance intention for several reasons. First, students are digital natives who exhibit high technology readiness and are among the earliest adopters of AI-powered health tools, making their usage patterns and continuance decisions indicative of future mainstream user behavior. Second, while students may have lower acute healthcare needs than older adults, they frequently use AI doctors for preliminary symptom checking, health information seeking, and wellness management—scenarios that mirror many real-world use cases. Third, understanding continuance intention among this population provides valuable insights for designing engaging AI healthcare services for younger generations, who will constitute the primary user base in the coming years. Nevertheless, we acknowledge the limitation of a student-dominated sample and discuss its implications in Section 6.4 (Tables 3, 4).

**Table 3:** Descriptive statistics of respondent characteristics.

Demographic variable		Size	%
Gender	Male	85	36.480
	Female	148	63.520
Age	< 18 years old	13	5.580
	18–30 years old	160	68.670
	30–40 years old	13	5.580
	40–50 years old	32	13.730
	> 50 years old	15	6.440
Education	Senior middle school or below	31	13.300
	Junior college	31	13.300
	Bachelor's degree	161	69.100
	Master's degree or above	10	4.290
Occupation	Students	157	67.380
	Corporate employee	27	11.590
	Public institution staff	11	4.720
	Government agency/Civil servant	5	2.150
	Entrepreneur/Self-employed	5	2.150
	Freelancer	24	10.300
	Other (please specify)	4	1.720
Medical-related background	Yes	23	9.870
	No	210	90.130
Monthly income	Students, no personal income	155	8.664
	5,000 RMB or less	31	15.523
	5,001–8,000 RMB	13	24.549
	8,001–15,000 RMB	9	30.686
	15,001–20,000 RMB	1	14.079
	More than 20,000 RMB	4	6.498

**Table 4:** Heterotrait-monotrait (HTMT) matrix.

Construct	AU	CT	CUI	EA	TA	TA × AU
AU						
CT	0.653					
CUI	0.785	0.785				
EA	0.529	0.545	0.890			
TA	0.179	0.384	0.317	0.461		
TA × AU	0.242	0.312	0.228	0.047	0.191	

## 5 Data analysis and results

### 5.1 Measurement model

In this study, SmartPLS 4.0 software was used to evaluate the measurement model and to test the reliability, convergent validity, and discriminant validity of the data. Specifically, reliability was evaluated using Cronbach's alpha, composite reliability (CR), and average variance extracted (AVE). As shown in Table 5, the values of Cronbach's alpha ranged from 0.784 to 0.892, exceeding the recommended threshold of 0.7; the composite reliability (CR) values ranged from 0.859 to 0.925, which are above the benchmark of 0.7; and the average variance extracted (AVE) values ranged from 0.605 to 0.756, all exceeding the required threshold of 0.5. These results indicate that all measurement indicators exhibit good reliability. Convergent validity and discriminant validity were measured using factor loadings and the square root of the AVE, respectively. According to the results, all factor loadings were above 0.7 at a significance level of *p* < 0.01 (see Table 6), demonstrating good convergent validity. Additionally, as shown in Table 5, the square root of the AVE for each construct was greater than its correlation coefficients with other constructs, thereby confirming discriminant validity.

**Table 5:** Descriptive statistics and inter-construct correlations.

Items	Alpha	rho_A	CR	AVE	AU	CT	CUI	EA	TA
AU	0.784	0.798	0.859	0.605	0.778				
CT	0.874	0.876	0.914	0.726	0.541	0.852			
CUI	0.892	0.893	0.925	0.756	0.666	0.694	0.869		
EA	0.888	0.892	0.923	0.750	0.461	0.476	0.791	0.866	
TA	0.855	0.858	0.902	0.697	0.142	0.333	0.278	0.404	0.835

AU, AI doctor use; CT, cognitive trust; CUI, continuance intention; EA, emotional attachment; TA, technology anxiety.

**Table 6:** Cross loadings.

Items	AU	CT	CUI	EA	TA
AU1	**0.768**	0.379	0.494	0.446	0.215
AU2	**0.727**	0.383	0.436	0.203	0.036
AU3	**0.832**	0.456	0.628	0.463	0.089
AU4	**0.780**	0.465	0.487	0.268	0.084
CT1	0.432	**0.829**	0.617	0.521	0.233
CT2	0.489	**0.899**	0.610	0.321	0.321
CT3	0.482	**0.844**	0.519	0.313	0.245
CT4	0.442	**0.834**	0.614	0.467	0.330
CUI1	0.581	0.656	**0.850**	0.640	0.250
CUI2	0.564	0.485	**0.883**	0.752	0.317
CUI3	0.602	0.656	**0.908**	0.700	0.241
CUI4	0.568	0.605	**0.834**	0.658	0.157
EA1	0.322	0.518	0.674	**0.793**	0.266
EA2	0.424	0.411	0.747	**0.910**	0.356
EA3	0.415	0.341	0.635	**0.887**	0.392
EA4	0.429	0.390	0.682	**0.869**	0.379
TA1	0.176	0.216	0.199	0.358	**0.795**
TA2	0.166	0.319	0.234	0.279	**0.816**
TA3	0.055	0.308	0.237	0.320	**0.851**
TA4	0.086	0.271	0.255	0.388	**0.875**

The bold values are loadings for each item that are above the recommended value of 0.5; and an item's loadings on its own variable are higher than all of its cross-loadings with other variable.

To further assess discriminant validity, the heterotrait-monotrait (HTMT) ratio of correlations was examined. As shown in Table 4, all HTMT values were below the conservative threshold of 0.90 (45), ranging from 0.047 to 0.890. Specifically, the HTMT value between cognitive trust and emotional attachment was 0.545, well below the recommended cutoff, confirming that the two constructs are empirically distinct. The HTMT value between emotional attachment and continuance intention was 0.890, which slightly exceeds the 0.85 threshold but remains below the more lenient threshold of 0.90 (46); this is theoretically acceptable given that emotional attachment is a strong direct predictor of continuance intention. Overall, the HTMT results provide additional evidence of adequate discriminant validity.

Given that single-source and cross-sectional data are prone to common method variance (CMV), this study employed Harman's single-factor test to evaluate the 20 scale items in our model to ensure that the dataset was free of CMV. The results showed that the highest covariance explained by a single factor was 47.948%, which fell below the cut-off value of 50% (47), indicating that common method bias does not pose a serious issue in this study. Second, the full collinearity variance inflation factor (VIF) approach was applied to further assess common method bias (48). All VIF values were below the conservative threshold of 3.3, ranging from 1.273 to 2.137, indicating that common method bias is not a serious concern in this study (46, 48). Furthermore, the standardized root mean squared residual (SRMR) was used to assess the goodness of fit for the saturated model. The SRMR value was found to be 0.064, which is lower than the suggested value of 0.08 (49), signifying that the model could be considered to have a good fit. Therefore, the proposed measurement model demonstrates satisfactory psychometric properties and is suitable for further structural analysis and hypothesis testing. The effect size analysis showed that AI use had a medium effect on cognitive trust (f^2^ = 0.308) and emotional attachment (f^2^ = 0.246). Predictive relevance was assessed using Stone–Geisser's Q^2^ values obtained through the blindfolding procedure. All endogenous constructs showed Q^2^ values greater than zero, indicating that the model has predictive relevance.

### 5.2 Structural model

**Figure 2:**
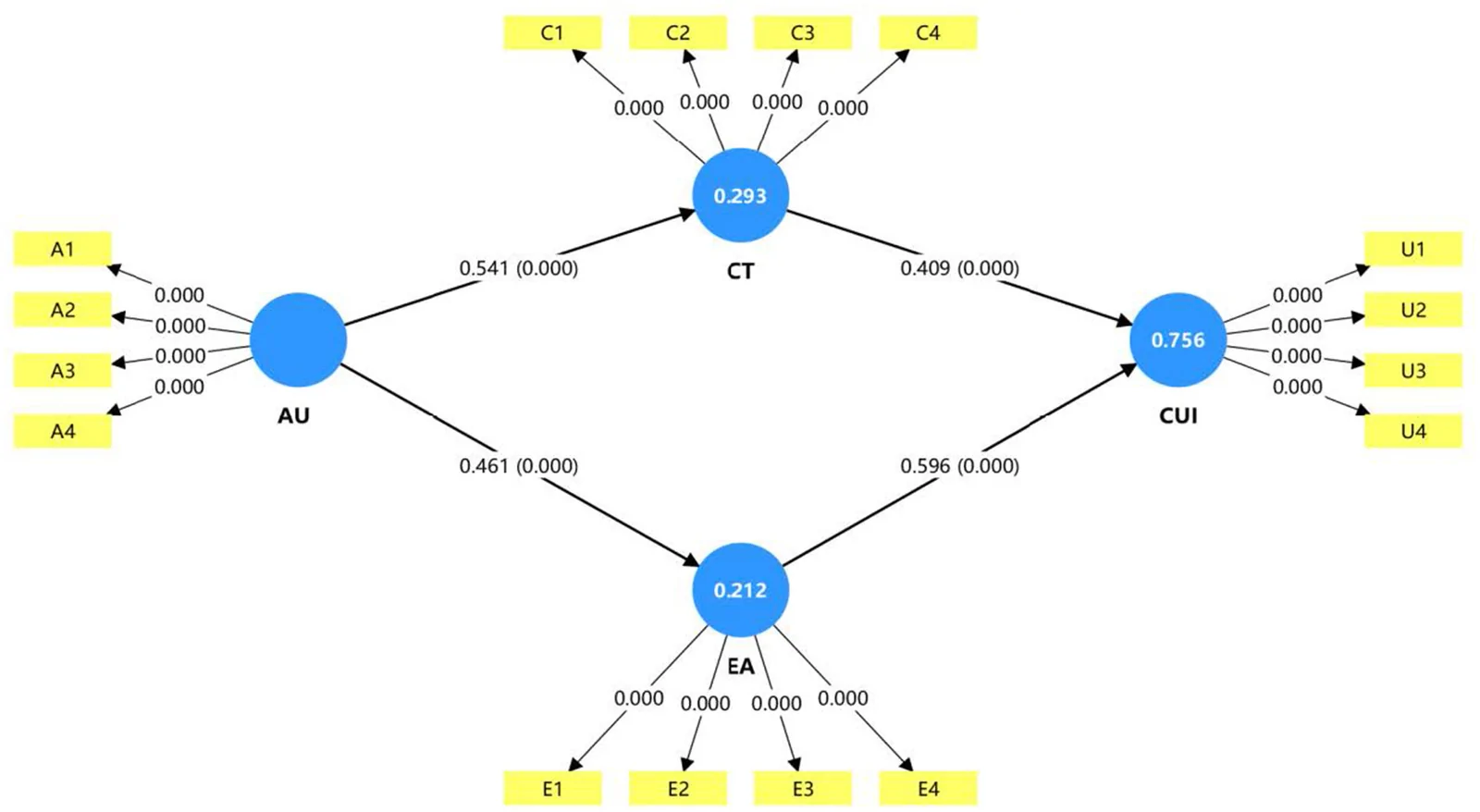
Results of the structural model analysis.

The results of the structural model analysis are shown in Figure 2. The findings support the main research hypotheses proposed in this study. First, AI doctor use has a significant positive effect on cognitive trust (β = 0.541, *p* < 0.001); therefore, hypothesis H1 is supported. This finding aligns with recent research confirming that trust serves as a key mechanism linking AI adoption to continued engagement. Second, AI doctor use also has a significant positive effect on emotional attachment (β = 0.461, *p* < 0.001), supporting hypothesis H2. This is consistent with studies showing that perceived anthropomorphism is associated with emotional attachment in AI Chatbots interactions (26). Regarding the paths influencing continuance intention, cognitive trust has a significant positive effect on it (β = 0.293), supporting hypothesis H3. Recent empirical evidence similarly demonstrates that perceived trust directly and positively associated with continuance intention. Notably, emotional attachment exhibits an even stronger positive effect on continuance intention (β = 0.409, *p* < 0.001), supporting hypothesis H4. This differential finding resonates with prior research indicating that emotional attachment exerts greater explanatory power on continuance intention than trust does (26). Taken together, these results indicate that users' continued engagement with AI doctors is associated with both their cognitive trust and emotional attachment, which in turn positively affects their intention to continue using the service. The proposed research model is effectively validated by the data.

### 5.3 Mediating effects

To examine the mediating roles of cognitive trust (CT) and emotional attachment (EA) in the relationship between AI use (AU) and continuance intention (CUI), the bootstrap method (5,000 resamples) was implemented using SmartPLS. The bootstrap approach employed here has been widely adopted by researchers. Two methods were applied, the bias-corrected method and the percentile method, and a 95% confidence level was selected for the confidence intervals. According to established guidelines, a mediating effect is considered statistically significant if the 95% confidence interval for the indirect effect does not include zero (50). Specific types of mediation effects were identified as follows: (a) when the confidence intervals of both the direct and indirect effects do not include zero, a partial mediation effect is present; (b) when the confidence interval of the indirect effect does not include zero, but the confidence interval of the direct effect does include zero, a full mediation effect is present. Table 7 shows our results. As shown in Table 7, both mediating pathways were significant. First, emotional attachment partially mediates the relationship between AI use and continuance intention. Second, cognitive trust also partially mediates the relationship between AI use and continuance intention. These findings indicate that AI use is not only directly associated with users' intention to continue using the service but also influences this relationship through both cognitive trust and emotional attachment.

**Table 7:** Results of mediating effects.

Path	Original sample (O)	Sample mean (M)	Lower CI	Upper CI	*P* values
AU → CT → CUI	0.221	0.228	0.109	0.361	0.001
AU → EA → CUI	0.275	0.281	0.158	0.406	0.000

Bootstrap resampling = 5000 times. AU, AI doctor use; CT, cognitive trust; EA, emotional attachment; CUI, continuance intention.

### 5.4 Moderating effects

To examine the moderating role of technology anxiety in the relationships between AI use and cognitive trust, as well as between AI use and emotional attachment, this study employed the product indicator approach within the PLS-SEM framework (51). The interaction terms between AI use and technology anxiety (TA × AU) were created for each path using the two-stage approach implemented in SmartPLS 4.0 (46). The structural model was then estimated with 5,000 bootstrap resamples to obtain standard errors and confidence intervals for the interaction effects.

**Figure 3:**
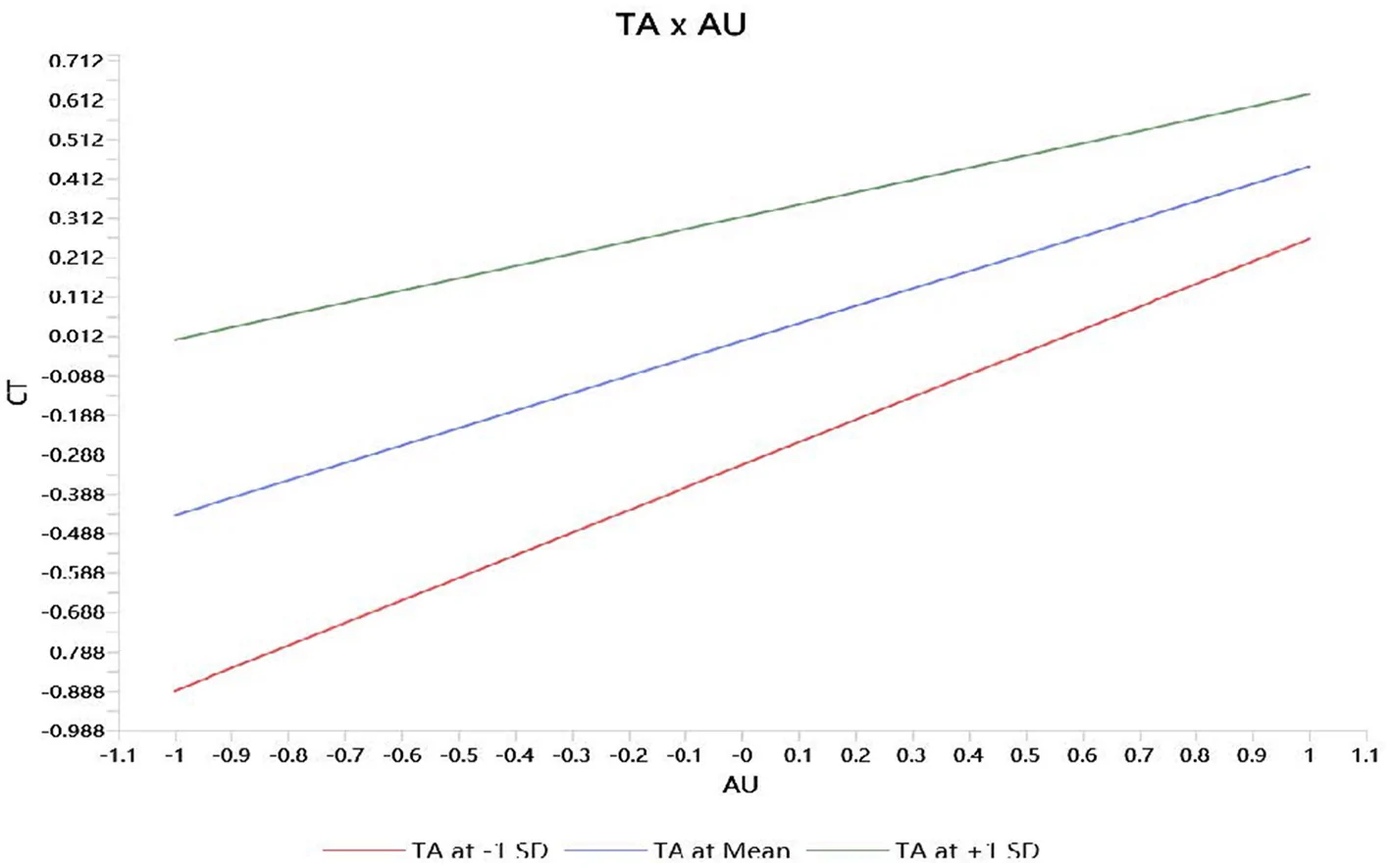
Moderating effect of technology anxiety on the relationship between AI use and cognitive trust.

**Figure 4:**
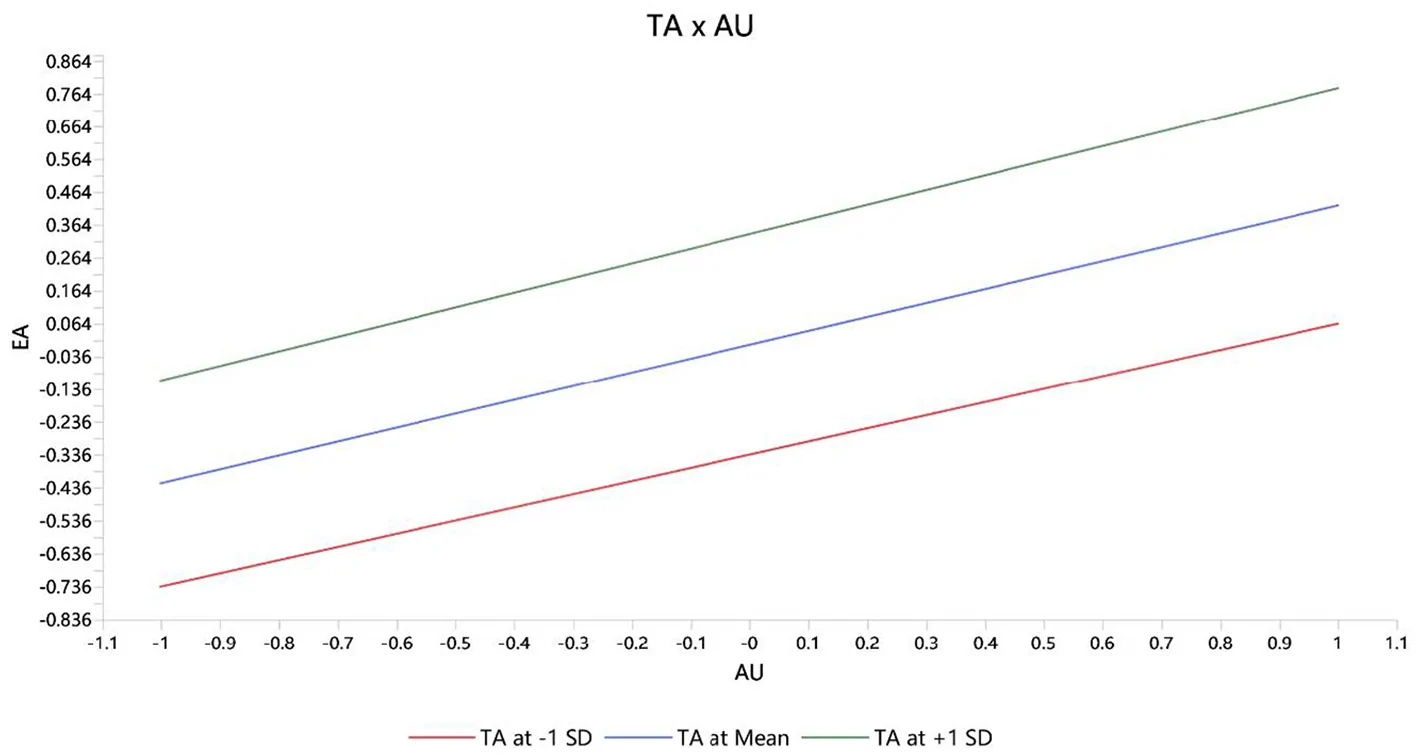
Moderating effect of technology anxiety on the relationship between AI use and emotional attachment (not supported).

The results, presented in Table 8, indicate that technology anxiety exerts a significant negative moderating effect on the relationship between AI use and cognitive trust (β = −0.131, t = 2.319, *p* < 0.05). This moderating effect is illustrated in Figure 3, where the positive relationship between AI use and cognitive trust becomes weaker as technology anxiety increases. Thus, Hypothesis H5 is supported. In contrast, the moderating effect of technology anxiety on the relationship between AI use and emotional attachment is not statistically significant (β = 0.023, t = 0.398, *p* > 0.05). Therefore, Hypothesis H6 is not supported (Figure 4). These findings are consistent with the theoretical expectation that anxiety primarily disrupts effortful, analytical processing (required for cognitive trust) while leaving automatic, experiential bonding (emotional attachment) relatively intact.

**Table 8:** Result of moderating effects analysis.

No	Hypothesized relationship	Path coefficient	T-Statistics	*P*-Values	Lower CI	Upper CI	Decision
1	TA × AU → CT	−0.131	2.319	0.020	−0.243	−0.023	Supported
2	TA × AU → EA	0.023	0.398	0.690	−0.105	0.118	Not supported

AU, AI doctor use; TA, technology anxiety; CI, confidence interval.

## 6 Conclusions

### 6.1 Key findings

First, regarding the direct effects among variables, the results demonstrate that AI use has a significant positive impact on both cognitive trust and emotional attachment. Furthermore, both cognitive trust and emotional attachment significantly enhance users' continuance intention. These findings align with prior research emphasizing the importance of trust and affective bonds in technology adoption and continuance (52, 53). However, while previous studies have largely examined trust as a unidimensional construct in general technology contexts, this study extends the literature by distinguishing between cognitive and emotional pathways specifically within the emerging domain of AI healthcare services. Recent research by Kyung and Kwon (3) similarly demonstrated that AI's deficient affective trust, rather than comparable cognitive trust, plays a decisive role in the acceptance of AI-based preventive health interventions. By revealing that emotional attachment exerts a stronger influence on continuance intention than cognitive trust, this study highlights the particularly salient role of affective engagement in health-related AI interactions—a nuance further supported by Howcroft et al. (12) meta-analysis, which found that AI Chatbots are frequently perceived as more empathic than human healthcare professionals in text-only scenarios, with a standardized mean difference of 0.87 favoring AI. The stronger influence of emotional attachment compared with cognitive trust highlights a distinctive characteristic of AI healthcare interactions. Unlike many conventional digital services where continued use is primarily driven by functional evaluations, healthcare decisions often occur under conditions of uncertainty, vulnerability, and emotional stress. In such situations, users seek not only accurate medical information but also psychological reassurance and a sense of being supported. Therefore, emotional attachment formed through repeated conversations, personalized responses, and perceived social presence may become a stronger driver of continued engagement than competence-based evaluations alone. This finding also provides a nuanced comparison with conventional healthcare relationships. In traditional doctor–patient relationships, trust is often primarily grounded in professional expertise, medical competence, and institutional credibility. However, AI healthcare Chatbots lack physical presence and formal professional identity, which may shift users' reliance from purely competence-based evaluation toward relationship-like experiences generated through interaction. Consequently, emotional attachment may play a particularly salient role in sustaining engagement with AI doctors. This pattern may also differ from other AI service contexts. In productivity-oriented or transactional AI applications, such as recommendation systems or workplace assistants, users' continued engagement is often determined primarily by efficiency, usefulness, and performance outcomes. In contrast, AI healthcare Chatbots operate in an emotionally sensitive domain where users' psychological needs are more prominent. Thus, emotional attachment may have greater explanatory power in healthcare AI than in other functional AI applications.

Second, concerning the mediating mechanisms, the findings reveal that both cognitive trust and emotional attachment serve as significant mediators in the relationship between AI use and continuance intention, with indirect effect values of 0.182 and 0.252, respectively. This indicates that AI use not only directly associated with users' continuance decisions but also operates indirectly by fostering rational confidence and emotional bonds. Notably, all mediation pathways through cognitive trust and emotional attachment were statistically significant, except for the pathway involving the interaction term TA × AU through emotional attachment. The non-significance of this specific indirect effect suggests that when users experience heightened technology anxiety, the emotional pathway from AI use to continuance intention remains relatively robust and less susceptible to interference. This finding aligns with recent research by Wang et al. (19), who found that emotional support from GAI doctors significantly associated with trust repair following service failures, suggesting that affective mechanisms may operate independently of cognitive appraisals. One possible explanation is that emotional attachment develops through repeated, habitual interactions that are less cognitively demanding, as Park et al. (54) demonstrated that anthropomorphic design cues in healthcare Chatbots influence use intention through the mediating role of trust, with emotional support having a significant positive impact on both affective and cognitive trust. In contrast, cognitive trust requires deliberate, analytical processing of system performance, making it more prone to the inhibiting effects of anxiety—a pattern consistent with Liu et al.'s (55) finding that anthropomorphism facilitates reuse intention through trust mechanisms that vary with contextual factors such as disease severity. Unlike previous studies that conceptualize trust in AI healthcare primarily as a multidimensional evaluation of system competence, reliability, or benevolence, this study distinguishes trust-related cognitive evaluation from relationship-based emotional attachment. This distinction extends prior research by showing that continued engagement with AI healthcare Chatbots is shaped not only by whether users believe AI is capable but also by whether they develop relational bonds with AI systems.

Third, with respect to the moderating effects, the results demonstrate that technology anxiety significantly moderates the relationship between AI use and cognitive trust, indicating that higher levels of anxiety weaken the positive effect of usage on rational trust. However, technology anxiety does not significantly moderate the relationship between AI use and emotional attachment. This asymmetric moderating effect suggests that anxiety primarily interferes with users' capacity to engage in deliberate, cognitive evaluation of system competence, while leaving the more automatic, affect-driven formation of emotional bonds relatively intact. This finding is consistent with the theoretical distinction between analytical processing (on which cognitive trust depends) and experiential processing (on which emotional attachment relies), confirming that anxiety selectively disrupts effortful cognition while sparing automatic affective bonding. This finding is consistent with recent research observing that emotional attachment in human-AI interaction tends to develop through intuitive, experiential processes that are less susceptible to cognitive disruptions such as performance ambiguity or perceived risk (56). Furthermore, contemporary studies on AI acceptance in healthcare have noted that users' affective bonds with conversational agents often persist despite technological anxieties, as these bonds are built on repeated positive interactions and perceived social presence rather than analytical evaluations (57). Schillaci et al. (25) similarly found that anthropomorphic Chatbots influence source credibility and user satisfaction through mechanisms that may operate independently of users' cognitive evaluations of system performance. Therefore, the non-significant moderation on the emotional pathway can be attributed to the relatively automatic and experience-based nature of emotional attachment formation, which relies less on conscious, analytical processing and more on cumulative, positive interactions over time—a pattern increasingly recognized in recent human-computer interaction literature (55). This asymmetric moderating effect provides an important theoretical insight: technology anxiety does not simply reduce all forms of AI-related psychological engagement. Instead, it selectively constrains cognition-based evaluations while leaving relationship-based emotional bonds relatively unaffected. This finding suggests that users may simultaneously experience uncertainty regarding AI competence and maintain emotional connections developed through previous interactions.

### 6.2 Theoretical implications

First, drawing upon cognitive-affective processing theory and trust-commitment framework, this study extends these theoretical lenses by integrating both cognitive and affective pathways into a unified model explaining users‘ continuance intention toward AI medical services. While prior research has predominantly relied on cognitive-based models such as the Technology Acceptance Model (TAM) and Expectation Confirmation Theory (ECT) to explain technology continuance, these frameworks largely emphasize rational evaluations of usefulness and satisfaction, often overlooking the emotional dimension of human-technology interaction. Recent systematic reviews have similarly critiqued this cognitive bias, noting that traditional adoption frameworks need to integrate psychosocial factors including emotional responses and trust to better explain healthcare technology acceptance (58). By incorporating both cognitive trust (representing the rational, competence-based evaluation) and emotional attachment (representing the affective, relationship-based bond) as dual mediators, this study enriches the theoretical understanding of how AI use translates into sustained engagement. Recent research by Zhang et al. (18) similarly demonstrated that in AI-based medical consultations, both cognitive empowerment and emotional empowerment significantly influence users' usage intentions, supporting the dual-pathway perspective. Specifically, this study reveals that emotional attachment exerts a stronger influence on continuance intention than cognitive trust, challenging the conventional emphasis on cognitive evaluations and highlighting the need to integrate affective mechanisms into mainstream technology adoption theories—particularly in healthcare contexts where interactions are inherently personal and emotionally charged.

Second, this study identifies and validates a dual-mediation mechanism, revealing that AI use influence continuance intention through two distinct psychological pathways: cognitive trust and emotional attachment. Although information systems scholars have extensively examined the multidimensional nature of trust, the majority of empirical studies still operationalize trust as a single, aggregated construct, obscuring the distinct mechanisms through which different trust dimensions operate (52). Furthermore, prior studies have rarely examined how these two pathways operate simultaneously and comparatively within the same model. By empirically demonstrating that both pathways significantly mediate the relationship, and that the emotional pathway carries a larger indirect effect, this study advances the literature by uncovering the complementary yet differentiated roles of cognitive and affective mechanisms. This finding challenges the conventional assumption that healthcare technology continuance is primarily driven by competence-based trust and suggests that relational experiences are increasingly important in AI-mediated healthcare. Notably, the finding that all mediation pathways through cognitive trust and emotional attachment were statistically significant, except for the pathway involving the interaction term TA × AU through emotional attachment, further refines our understanding of how these pathways diverge under contextual influences. This nuanced finding is consistent with Chen et al.'s (19) research on trust repair in GAI doctors, which revealed that emotional support significantly associated with trust repair following service failures, suggesting that affective mechanisms may operate independently of cognitive appraisals. The non-significance of the specific indirect effect through emotional attachment under high anxiety conditions suggests that emotional pathways may be more resilient—a pattern that extends our theoretical understanding of how different psychological mechanisms respond to user apprehensions.

Third, this study introduces technology anxiety as a critical boundary condition and reveals its differential moderating effects across the cognitive and affective pathways. Although existing literature has regarded technology anxiety as a barrier to initial adoption (27), limited attention has been paid to how it differentially shapes the ongoing psychological processes that sustain use. By demonstrating that technology anxiety significantly attenuates the relationship between AI use and cognitive trust, but does not significantly moderate the relationship between AI use and emotional attachment, this study reveals an important theoretical nuance: anxiety selectively disrupts cognitive, analytical processing while leaving affect-driven, experiential bonding relatively intact. This asymmetric moderating effect extends cognitive-affective processing theory (59) by specifying the differential vulnerability of cognitive vs. affective pathways to negative emotional states. The non-significant moderation on the emotional pathway can be attributed to the relatively automatic and experience-based nature of emotional attachment formation, which relies less on conscious, analytical processing and more on cumulative, positive interactions over time. This finding challenges the implicit assumption in prior research that barriers such as anxiety uniformly weaken technology adoption processes, instead revealing a more complex pattern where different psychological mechanisms exhibit varying degrees of resilience—a nuance further supported by Liu et al.'s (60) research on Gen AI adoption, which identified fear of AI as a negative moderating factor that weakens direct effects of technology attributes on intention, emphasizing the critical role of individual characteristics in shaping human-AI interactions.

Beyond the dual-pathway model, this study advances a higher-order theoretical proposition: in AI healthcare contexts, users develop anxiety-resistant affective bonding with AI doctors. Unlike cognitive trust, which is vulnerable to technology-induced apprehension, emotional attachment operates through habitual, experience based processes that are largely automatic and require minimal cognitive resources. This insight challenges the conventional assumption that user anxiety uniformly undermines technology acceptance. Instead, it suggests that AI healthcare systems can sustain user engagement by fostering emotional bonds that are resilient to negative affective states—a mechanism we term affective resilience (61). Furthermore, our findings imply a potential substitution effect between cognitive and emotional pathways: when cognitive trust is compromised by high technology anxiety, emotional attachment can partially compensate, maintaining continuance intention through a distinct route. This emotional substitution mechanism extends existing theories of technology continuance, which have predominantly emphasized cognitive evaluations (3). Practically, it suggests that designing for emotional connection may be a more robust strategy than merely improving system transparency or reliability, especially for users prone to technology anxiety.

### 6.3 Practical implications

First, the finding that AI use positively associated with both cognitive trust and emotional attachment, which in turn drive continuance intention, provides important guidance for designing effective user engagement strategies. On the one hand, to strengthen the cognitive pathway, practitioners should focus on enhancing the transparency, reliability, and explain ability of AI medical services. Specifically, AI systems should provide clear, evidence-based explanations for their diagnostic suggestions, cite authoritative medical sources when appropriate, and present information in a way that users can easily understand and verify. These features help users develop rational confidence in the system's professional competence, thereby increasing their willingness to continue using the service. This aligns with recent research by Kyung and Kwon (3), who found that algorithmic transparency and the combination of AI with human expert opinions can significantly improve the effectiveness of AI-based preventive health interventions. On the other hand, to activate the emotional pathway, designers should incorporate affective elements into the user interface, such as empathetic conversational styles, personalized responses based on interaction history, and warm, human-like communication tones. These design choices can transform purely transactional interactions into emotionally meaningful experiences, fostering a sense of closeness and habitual reliance that strongly predicts long-term engagement. Given that emotional attachment exerts a stronger total effect on continuance intention than cognitive trust, healthcare organizations should place particular emphasis on cultivating users' affective bonds with AI systems, recognizing that in health-related contexts—where interactions often involve vulnerability and personal concern—emotional connections may matter more than purely rational assessments. This finding is consistent with Kyung and Kwon (3) observation that AI's deficient affective trust, rather than comparable cognitive trust, plays a decisive role in the acceptance of AI-based health interventions. Furthermore, AI developers should design healthcare Chatbots that balance technical competence with relational capability. Transparent explanations of AI-generated recommendations can improve cognitive trust, whereas personalized conversations and empathetic responses can foster emotional attachment. Moreover, integrating human escalation mechanisms, such as allowing users to transfer complex cases to professional healthcare providers, may reduce perceived risk and increase users' willingness to continue using AI healthcare services. While emotional attachment can enhance users' continued engagement with AI healthcare Chatbots, excessive emotional dependence may also create potential ethical concerns. Users may over-rely on AI systems for healthcare decisions, underestimate system limitations, or substitute AI interactions for necessary professional medical consultation. Therefore, AI healthcare providers should design mechanisms that encourage appropriate emotional engagement while maintaining clear boundaries between supportive interaction and professional healthcare relationships. Features such as transparency about AI capabilities, reminders of system limitations, and human escalation mechanisms can help ensure that emotional attachment supports rather than compromises responsible healthcare usage.

Second, the moderating role of technology anxiety and the differential patterns observed across the two mediating pathways offer nuanced insights for targeted intervention strategies. The finding that technology anxiety significantly weakens the relationship between AI use and cognitive trust suggests that anxious users require additional support to translate their usage experiences into rational confidence. For this user segment, practitioners should implement anxiety-reduction measures throughout the user journey. During initial onboarding, guided tutorials, simplified interfaces, and clear privacy and security assurances can help lower the psychological barriers that inhibit trust formation. During ongoing use, features such as real-time assistance buttons, options for human verification of AI suggestions, and transparent communication about system limitations can provide anxious users with a safety net that facilitates gradual trust building. This approach is supported by recent research by Aisyah et al. (62), who found that AI configuration and design elements can effectively mitigate the impact of social-technical blindness and job replacement anxiety among healthcare professionals, suggesting that thoughtful system design can alleviate technology-related apprehensions. Furthermore, Svestkova et al. (4) demonstrated that trust serves as a critical mediator in the adoption of generative AI for health purposes, with health anxiety influencing willingness to use AI only indirectly through trust—underscoring the importance of building trust as a pathway to engage anxious users. AI healthcare providers should adopt differentiated strategies based on users' technology anxiety levels. For users with high technology anxiety, organizations should focus on reducing cognitive barriers and uncertainty. This can be achieved through simplified interface designs, transparent explanations of AI decision processes, and step-by-step guidance during interactions. Providing clear information about how AI generates recommendations and allowing users to understand system limitations may alleviate anxiety and strengthen confidence in AI healthcare services. For users with low technology anxiety, healthcare organizations should emphasize relational enhancement rather than merely improving functional performance. Personalized recommendations, conversational continuity, and emotionally supportive communication can strengthen users' emotional attachment to AI healthcare Chatbots and encourage continued engagement.

### 6.4 Limitations and future research

This study has several limitations. First, although the proposed model specifies directional relationships based on established theories, the cross-sectional research design limits our ability to draw definitive causal conclusions (63, 64). Future research could employ longitudinal designs, field experiments, or experimental approaches to further validate the causal relationships among AI use, cognitive trust, emotional attachment, and continuance intention. Second, our sample was collected from AI healthcare Chatbot users in China. Although China provides an appropriate context due to the rapid development of digital healthcare services, cultural differences in technology acceptance, healthcare systems, and doctor–patient relationships may influence users' perceptions of AI healthcare Chatbots. Therefore, future research should replicate this model in different cultural and healthcare contexts to examine its generalizability (65). Third, self-reported data may be subject to social desirability effects despite statistical tests (66). Fourth, the model excluded potentially important variables like health literacy, privacy concerns, and individual differences. Fifth, rapid AI evolution means findings based on current systems may not apply to future iterations (67). Sixth, the sample exhibits a demographic skew, with university students comprising 67.38% of the respondents and the majority of participants aged 18–30 years. Although students are relevant early adopters of AI health tools (as discussed in Section 4.2), this composition may limit the generalizability of our findings to broader populations—particularly older adults, individuals with chronic conditions, or those with lower digital literacy, who may have different patterns of trust formation and emotional attachment. Finally, although this study identifies cognitive trust and emotional attachment as important mechanisms underlying AI healthcare Chatbot continuance intention, other healthcare-specific factors were not incorporated into the current model. In particular, privacy concerns, perceived risks, medical reliability, and perceived competence may play important roles in shaping users' trust evaluations and continuance decisions. Future research could extend our framework by incorporating these factors to provide a more comprehensive understanding of AI healthcare adoption and continued engagement. Future research should also validate the proposed model using more representative samples that include a wider age range, diverse occupational backgrounds, and actual patients with ongoing healthcare needs (74, 75). Additionally, subgroup analyses (e.g., students vs. non-students) or robustness checks controlling for demographic variables could further strengthen external validity. Future research should address these limitations by employing longitudinal designs to establish causality, (76) conducting cross-cultural comparisons, integrating behavioral logs with qualitative data, expanding theoretical models to include additional variables, focusing on specialized healthcare domains, and investigating how advances in generative and emotional AI reshape user engagement mechanisms.

## Data Availability

The data analyzed in this study is subject to the following licenses/restrictions: The data that support the findings of this study are available from the corresponding author upon reasonable request. Requests to access these datasets should be directed to Feifei Hao, aifeifei0210@126.com.
