# Correction: Trusting and staying with AI doctors: cognitive and emotional pathways driving continuance intention toward AI healthcare chatbots

**DOI:** 10.3389/fpubh.2026.1998933

**Published:** 2026-09-25

**Authors:** Zhanyou Wang, Haoran Han, Dongmei Han, Liang Ma, Feifei Hao

**Affiliations:** 1 School of Business Administration, Shandong Management University, Jinan, China; 2 School of Labor Relations, Shandong Management University, Jinan, China; 3 School of Information Engineering, Shandong Management University, Jinan, China; 4 School of Management Science and Engineering, Shandong University of Finance and Economics, Jinan, China; 5 College of Traditional Chinese Medicine, Shandong University of Traditional Chinese Medicine, Jinan, China

**Keywords:** AI doctor use, cognitive trust, continuance intention, emotional attachment, technology anxiety

Affiliation “School of Labor Relations, Shandong Management University, Jinan, China” was omitted from the paper. This affiliation has now been added for author Haoran Han.

Affiliation “School of Business Administration, Shandong Management University, Jinan, China” was erroneously added for author Haoran Han. This affiliation has now been removed for author Haoran Han.

The original version of this article has been updated.

